# Maternal voice and preterm infants development

**DOI:** 10.1186/1824-7288-41-S1-A14

**Published:** 2015-09-24

**Authors:** Giancarlo Gargano, Francesca Nuccini

**Affiliations:** 1 Neonatologia e Terapia Intensiva Neonatale, Arcispedale S. Maria Nuova - IRCCS, Reggio Emilia, Italy

## 

Mother's voice seems to have an important role in neurological development of the fetus and the newborn. 

Numerous studies have shown that the fetus perceives sounds and reacts to them since 26th–28th week of gestation and that he has the ability to discriminate between the maternal voice and other voices, showing a marked preference to the first [1,2]. 

The fetus within the uterus is in a sound environment, called “noise floor”, resulting from the combination of “internal noise”, such as the sound of maternal heartbeat, breathing and gastrointestinal activity, and “external noise”, principally the mother's voice.

Premature birth abruptly stops prenatal learning experiences and it causes a sudden transition from the quiet and lovely environment of the maternal womb, towards the noisy world of the NICU, often hostile and aggressive [3]. During the months in NICU, the baby is deprived of the biological maternal sounds and this could interfere with his neurodevelopment, in particular speech and language acquisition.

The Developmental Care programs aim to provide an extra-uterine environment similar to the maternal womb: control of light, noise, pain, postural care, kangaroo mother care are examples. Few studies addressed the beneficial effects of the early exposure to the mother's voice and recently some researches have shown that this sound can increase cardiorespiratory stability and growth, improve deep sleep, and shorten length of hospital stay [4-8].

In 2013 Loewy [9], using several acoustic stimuli, has shown that exposure to “intrauterine” stimuli and in particular to the mother's voice, meant an increased attention span and alertness, associated with a marked reduction in heart rate, increased stability of behavioral states and sleep quality and improved nutritional behavior and caloric intake .

Chorna et al. [10], using mother's voice played through a Pacifier-Activated Music player (PAM) during non-nutritive sucking, have demonstrated an improvement of the development of sucking ability and oral feeding skills in preterm infants.

Finally Picciolini et al. [3] have shown that early exposure to maternal voice, administered by bone conduction, according Tomatis’ method, exerts a beneficial effect on preterm infants autonomic system and neurobehavioral development. They have found a better neurofunctional assessment score at 3 months of corrected age vs a control group.

In conclusion, studies show encouraging results about mother's voice ability to promote optimal neurological development of preterm babies. We are studying the effects of the voice on mother-child attachment and its correlations with neurodevelopment outcomes.
